# Advertising Alternative Cancer Treatments and Approaches on Meta Social Media Platforms: Content Analysis

**DOI:** 10.2196/43548

**Published:** 2023-05-31

**Authors:** Marco Zenone, Jeremy Snyder, Jean-Christophe Bélisle-Pipon, Timothy Caulfield, May van Schalkwyk, Nason Maani

**Affiliations:** 1 Faculty of Public Health and Policy London School of Hygiene and Tropical Medicine London United Kingdom; 2 Faculty of Health Sciences Simon Fraser University Burnaby, BC Canada; 3 Health Law Institute University of Alberta Edmonton, BC Canada; 4 Global Health Policy Unit University of Edinburgh Edinburgh United Kingdom

**Keywords:** cancer, advertising, misinformation, false hope, Meta, Facebook, Instagram, Messenger, social media, exploitation, infodemiology, cancer treatment, online health information

## Abstract

**Background:**

Alternative cancer treatment is associated with a greater risk of death than cancer patients undergoing conventional treatments. Anecdotal evidence suggests cancer patients view paid advertisements promoting alternative cancer treatment on social media, but the extent and nature of this advertising remain unknown. This context suggests an urgent need to investigate alternative cancer treatment advertising on social media.

**Objective:**

This study aimed to systematically analyze the advertising activities of prominent alternative cancer treatment practitioners on Meta platforms, including Facebook, Instagram, Messenger, and Audience Network. We specifically sought to determine (1) whether paid advertising for alternative cancer treatment occurs on Meta social media platforms, (2) the strategies and messages of alternative cancer providers to reach and appeal to prospective patients, and (3) how the efficacy of alternative treatments is portrayed.

**Methods:**

Between December 6, 2021, and December 12, 2021, we collected active advertisements from alternative cancer clinics using the Meta Ad Library. The information collected included identification number, URL, active/inactive status, dates launched/ran, advertiser page name, and a screenshot (image) or recording (video) of the advertisement. We then conducted a content analysis to determine how alternative cancer providers communicate the claimed benefits of their services and evaluated how they portrayed alternative cancer treatment efficacy.

**Results:**

We identified 310 paid advertisements from 11 alternative cancer clinics on Meta (Facebook, Instagram, or Messenger) marketing alternative treatment approaches, care, and interventions. Alternative cancer providers appealed to prospective patients through eight strategies: (1) advertiser representation as a legitimate medical provider (n=289, 93.2%); (2) appealing to persons with limited treatments options (n=203, 65.5%); (3) client testimonials (n=168, 54.2%); (4) promoting holistic approaches (n=121, 39%); (5) promoting messages of care (n=81, 26.1%); (6) rhetoric related to science and research (n=72, 23.2%); (7) rhetoric pertaining to the latest technology (n=63, 20.3%); and (8) focusing treatment on cancer origins and cause (n=43, 13.9%). Overall, 25.8% (n=80) of advertisements included a direct statement claiming provider treatment can cure cancer or prolong life.

**Conclusions:**

Our results provide evidence alternative cancer providers are using Meta advertising products to market scientifically unsupported cancer treatments. Advertisements regularly referenced “alternative” and “natural” treatment approaches to cancer. Imagery and text content that emulated evidence-based medical providers created the impression that the offered treatments were effective medical options for cancer. Advertisements exploited the hope of patients with terminal and poor prognoses by sharing testimonials of past patients who allegedly were cured or had their lives prolonged. We recommend that Meta introduce a mandatory, human-led authorization process that is not reliant upon artificial intelligence for medical-related advertisers before giving advertising permissions. Further research should focus on the conflict of interest between social media platforms advertising products and public health.

## Introduction

Social media is both a valuable resource and a challenging arena for cancer patients and their families to navigate. Patients with cancer can find community [1,2], support [3,4], identity [5], and resources [6] across social media groups, pages, and forums. Social media also allows patients with cancer and their families to share updates and appeal for support within their networks [7,8]. Simultaneously, the internet contains widespread misinformation [9-14] about cancer, including its causes, evidence-based cancer treatments, and purported cancer treatments represented as efficacious when little, no, or disproven evidence exists for its use [15,16]. Nonetheless, content and articles with cancer misinformation shared on social media receive more engagement than factual sources [17].

Cancer misinformation reaches patients on social media and may have negative consequences, such as misinformed treatment decisions, worsened clinician-patient dynamics, and damaged caregiver-patient relationships [18,19]. In some cases, cancer misinformation can lead to patients with treatable or early-stage cancers opting out of evidence-based treatments in preference for alternative cancer treatments [20]. In other cases, patients with advanced cancers or limited treatment options may reasonably want to exhaust all options in search of a cure or to prolong life, leading them to try unproven, experimental, or alternative cancer therapies against their medical provider’s recommendation [21,22]. Patients who distrust health care, lack health literacy, do not have their informational needs met [23-25], and those with lower educational attainment are the most susceptible to cancer misinformation [26]. Alternative cancer treatment in patients with treatable or terminal cancer is associated with a reduced time to death than in patients with cancer undergoing scientifically supported treatment [27,28].

Compounding the misinformation difficulties faced by patients with cancer, alternative cancer treatment providers are alleged to actively promote unproven, experimental, and potentially harmful treatments [29,30]. Promotion occurs through various mediums and strategies, including websites and social media. Facebook groups, which can support patients with cancer through community and shared experiences, are targeted by posts advertising alternative cancer treatments or products [31]. Providers make unsubstantiated health claims, share disinformation [32], and distort the scientific evidence supporting their services in promotional activities [15]. The marketing of cancer treatments, especially by alternative providers, is harmful in that it provides false hope, utilizes medical resources inappropriately, and disrupts doctor-patient relationships [33]. The US Food and Drug Administration (FDA) regularly issues warnings to companies and services promoting unproven cancer products and treatments. In 2018, the FDA warned 14 alternative providers for “fraudulently claiming to diagnose, treat, or cure cancer,” with some selling and promoting their products on Facebook and Instagram [34]. However, warnings typically lead to limited negative consequences for providers.

While it is understood that advertising by alternative cancer providers is a source of harmful misinformation, an important area yet to be explored is how alternative cancer treatment providers utilize paid social media advertising products and tools to market their services. As opposed to other types of nondigital direct-to-consumer and nonpaid social media promotional activities or strategies [35-37] (eg, hosting a Facebook page without paid advertisements), targeted advertisements are uniquely effective at reaching specific groups via tailored messaging with little cost. Social media advertisers can target users in a certain age group, gender, geographic area, and income group, as well as people who demonstrate specific interests [38]. Advertisers can also employ advanced targeting features such as “custom” [39] or “lookalike” [40] audiences for further in-depth advertisement audience targeting. Applying social media–targeted advertising strategies for alternative cancer treatments may enable potential advertisers to target demographic groups fitting their target demographic profile, such as groups at a statistically higher risk of cancer or high-income earners. Targeted advertising may also enable advertisers to focus on demographics with “interests” or social activities demonstrating a higher likelihood of receptivity to their services (eg, “natural products”). Meta banned certain detailed terms (eg, “cancer”) to target as interests on January 19, 2022 [41]. However, as an advertiser, it is still possible to target the followers of known proponents of alternative medicine, such as Gwyneth Paltrow [42]. In summary, social media advertisements can reach and track a large, defined audience with little investment and effort.

To prevent the misuse of advertising tools, social media platforms require advertisers’ adherence to their platform-specific health-related advertising policies [43]. For example, Meta’s advertising policy states that “ads must not contain deceptive, false or misleading claims…that set unrealistic expectations for users.” Despite policies against misleading or harmful health advertising, Meta advertising tools promote scientifically unsupported public health messages and unproven products or services. Past research has found that paid Meta advertisements disseminated antivaccine [44] and protobacco content [45]. Patients with cancer have shared anecdotes of how they started to see advertisements for fake cancer cures after their diagnosis [18,29]. As recently as June 2022, paid advertisements for scientifically unsupported cancer treatments were reported on Meta platforms [46]. The current context suggests an immediate need to investigate the extent of alternative and unproven cancer treatment advertisements on Meta social platforms.

In this study, we partially address this need by systematically analyzing the advertising activities of prominent alternative cancer treatment practitioners on Meta platforms, including Facebook, Instagram, Messenger, and Audience Network. We specifically sought to determine (1) whether alternative cancer treatment paid advertising occurs on Meta social media platforms, (2) the strategies and messages alternative cancer providers use to reach and appeal to prospective patients, and (3) how the efficacy of alternative treatments is portrayed. Analyzing the advertising activities of alternative cancer treatment providers serves as a useful case study to examine Meta’s advertising infrastructure and its role in the propagation of misinformed cancer treatments.

## Methods

### Identifying and Retrieving Advertisements

To identify alternative cancer advertisements, we searched the Meta Ad Library [47]—a publicly accessible database of current advertisements running on Facebook, Instagram, Messenger, or Audience Network—by well-known alternative cancer providers to determine if marketing was occurring. We identified prominent alternative providers from a patient directory of nontraditional cancer clinics [48] and treatment destinations identified from a study investigating alternative cancer treatment crowdfunding [22]. The first source, Heal Navigator, is a website specializing in providing information on alternative treatment clinics outside of conventional care options. We chose this source because prospective patients and their families may use similar directories when researching alternative care options. The second source was a research study that investigated the crowdfunding activities of patients with cancer seeking complementary and alternative cancer treatment options. The study developed a list of 110 complementary and alternative cancer treatments, searched each treatment with the term “cancer” on GoFundMe, and subsequently collected the frequency of specific treatments being crowdfunded and the names of each alternative cancer clinic where patients sought to receive treatment. We chose this source because it reflects a novel data source to understand where patients are seeking to receive alternative cancer treatment. We considered “alternative cancer treatments” to include any cancer-specific treatment that is not medically supported, disproven, unproven, experimental, or in an early stage of research outside of a registered clinical trial or provided by an oncology trial unit. We identified 86 clinics to search for evidence of marketing alternative cancer treatments on a Meta social media platform.

Between December 6, 2021, and December 12, 2021, we visited each clinic’s unique advertising page daily on the Meta Ad Library and collected active advertisements. The information collected included the advertisement identification number, advertisement URL, date retrieved, active/inactive status, dates launched/run, advertiser page name, and a screenshot (image) or recording (if containing a video) of the advertisement. We collected 383 advertisements from 17 alternative cancer providers. To determine inclusion in the study, the first author (MZ) reviewed each advertisement to determine if the advertisement directly or indirectly offers an alternative, experimental, disproven, or unproven cancer treatment or approach to prospective patients with cancer through a paid Meta product advertisement. TC reviewed 50% of the inclusion decisions to ensure consistency in the inclusion criteria application. In total, we marked 310 advertisements for inclusion.

### Ethical Considerations

This study did not require ethics approval because all data collected were publicly available.

### Content Analysis

We conducted a content analysis [49] to analyze how alternative cancer providers communicate the benefits of their services through advertisements on Meta platforms. Content analysis has been used to study cancer content on numerous social media platforms [50-53] and is useful to observe, systematically categorize, and quantify communication message strategies and characteristics [54]. Authors MZ, JS, JCBP, and TC independently reviewed between 77 and 78 (25%) advertisements and met to determine pattern observations and identify key thematic frames. The authors developed an initial coding frame, and MZ test coded the advertisements. MZ then consulted with authors NM and MvS for their input into the coding frame. After minor modifications and similar code grouping, MZ coded the advertisements on the mixed methods software analysis program Dedoose (University of California, Los Angeles). We identified 8 advertising strategies. We also coded for the treatments mentioned and evaluated how alternative cancer providers portrayed treatment efficacy. When assessing efficacy representation, we chose to have another author review each statement for application consistency due to potential subjective interpretations of being cured or having one’s life prolonged. Author MvS reviewed efficacy statement coding decisions and agreed on 93% of efficacy code applications. Authors MZ and MvS then resolved disagreements through open discussion.

## Results

We identified 310 paid advertisements from 11 alternative cancer clinics on Meta (Facebook, Instagram, or Messenger) marketing alternative treatment approaches, care, and interventions. The clinic profiles of those hosting advertisements are summarized in Table 1. The clinics found in our study were in the United States (n=4, 36.4%), Mexico (n=4, 36.4%), Spain (n=2, 18.2%), and Thailand (n=1, 9.1%). Clinics may offer services in multiple locations. An expanded table detailing the treatments offered at each clinic and their treatment provider qualifications according to their websites is available in Multimedia Appendix 1.

**Table 1:** Clinic profile overview of alternative cancer treatment providers.

Clinic name	Total advertisements, n (%)	Location
Brio-Medical	146 (47.1)	Scottsdale, Arizona
Conners Clinic	44 (14.2)	Lake Elmo, Minnesota
CHIPSA^a^ Hospital	34 (11)	Tijuana, Mexico
Verita Life	23 (7.4)	Bangkok, Thailand
Budwig Center	14 (4.5)	Málaga, Spain
Immucura	14 (4.5)	Málaga, Spain
Hope4Cancer Treatment Centers	12 (3.9)	Tijuana, Mexico
Immunity Therapy Center	12 (3.9)	Tijuana, Mexico
Envita Medical Centers	6 (1.9)	Scottsdale, Arizona
Dayspring Cancer Clinic	4 (1.3)	Scottsdale, Arizona
Issels Immuno-Oncology	1 (0.3)	Tijuana, Mexico

^a^CHIPSA: Centro Hospitalario Internacional del Pacifico, SA.

**Figure 1:**
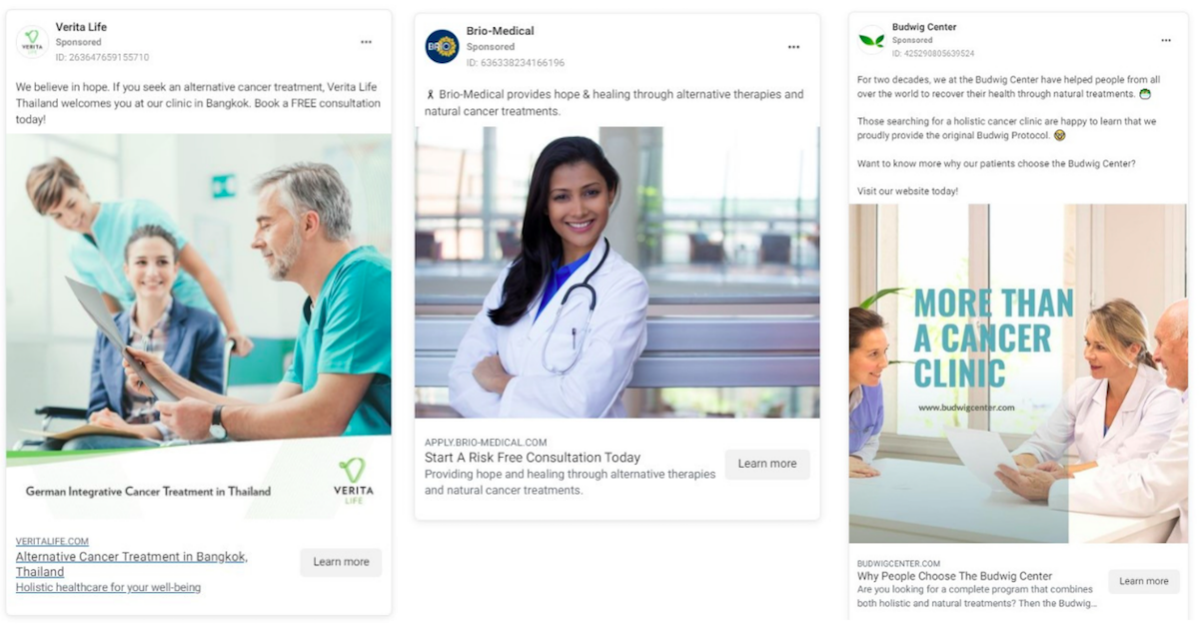
Advertisements depicting alternative cancer treatment provider is qualified to advise and administer cancer treatment.

**Figure 2:**
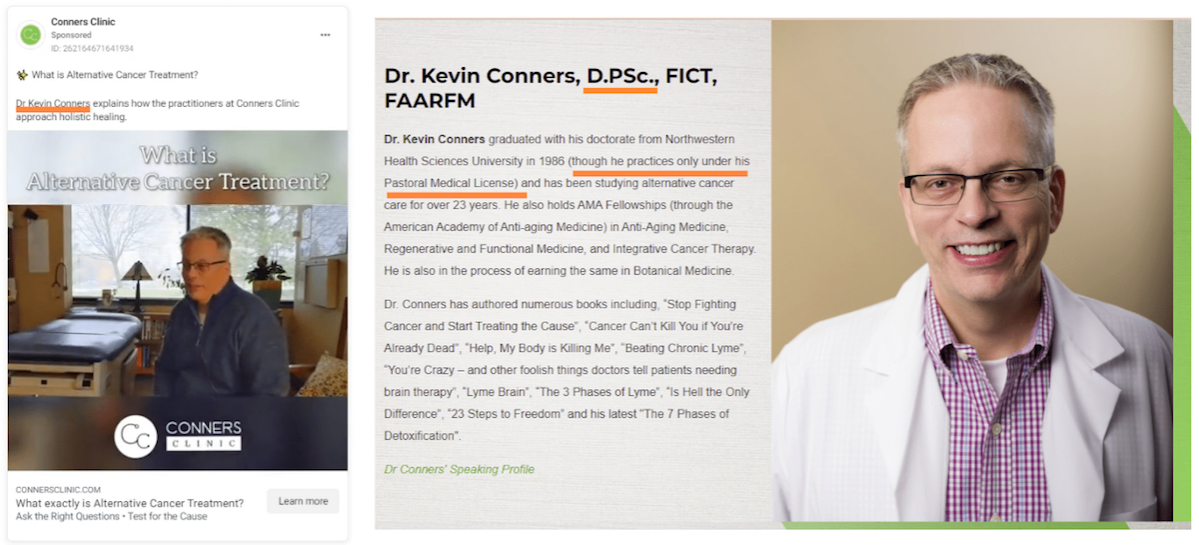
An example of an alternative cancer treatment advertisement depicting that the provider is qualified to advise cancer treatment with a website screenshot of provider qualifications.

Nearly all (n=289, 93.2%) advertisements featured imagery or text signifying that the provider is a qualified medical expert and may legitimately advise on and administer cancer treatment. Visual cues included images or text mentioning qualified health care providers (eg, doctors, surgeons), reference to interventions (treatment, medications, intravenous administration, therapies), medical imagery and equipment, and labeling the provider location as a “medical treatment center,” “clinic,” or related terms. Many clinics had staff providers with credentials that were not associated with expertise in primary cancer care or were legally barred from recommending primary cancer treatment. This included naturopaths, chiropractors, and other alternative medicine practitioners. Despite representation as a legitimate medical option, certain providers’ websites specify that they do not offer medical advice. Figure 1 displays illustrative examples of clinics presenting themselves as qualified cancer care and treatment providers. Figure 2 depicts an advertisement from Conners Clinic where the primary service provider refers to himself as “Dr” in a medical context giving cancer treatment advice. However, according to the Conners Clinic website [55,56], he practices under a “Pastoral Medical License” and does not offer medical advice.

**Figure 3:**
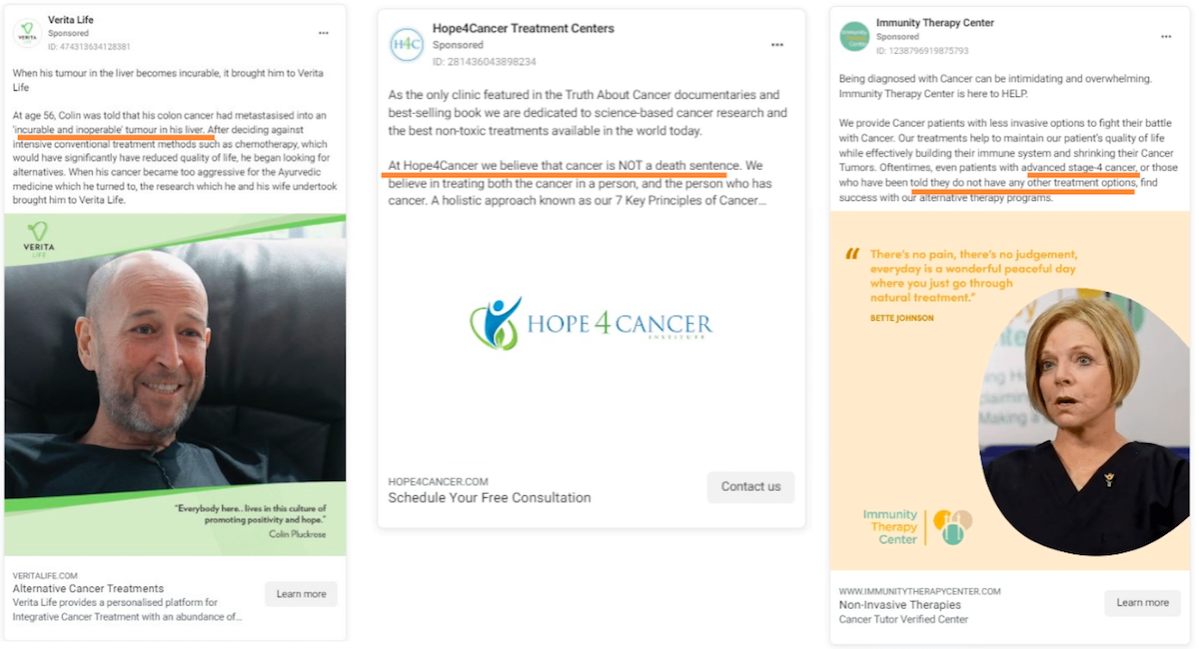
Advertisements appealing to persons with limited or no treatment options due to an advanced or terminal cancer prognosis.

In 65.5% (n=203) of the advertisements, providers appealed to persons with limited treatment options due to an advanced, aggressive, or terminal cancer prognosis to not give up seeking treatment because other “effective” options exist. Clinic advertisements invoked skepticism regarding noncurable cancer cases and gave examples of their alleged “success” in treating or curing terminal cancer cases. A CHIPSA (Centro Hospitalario Internacional del Pacifico, SA) hospital advertisement states their client “was told it [their cancer] was noncurative. But nearly 2 years after her initial diagnosis, and treatment at CHIPSA, she is cancer free.” Many advertisements invoked direct skepticism toward other health providers labeling a patient’s cancer as noncurative. In other cases, clinics offered examples where past clients were allegedly abandoned by their medical teams once their cancer reached an advanced or noncurative form. For example, an advertisement states, “She [patient] was ‘dropped’ by her doctors, put on hospice, and given only months to live. [Patient] and her husband [names redacted] refused this death sentence and ventured to CHIPSA Hospital in Mexico.” Illustrative screenshots are shown in Figure 3.

**Figure 4:**
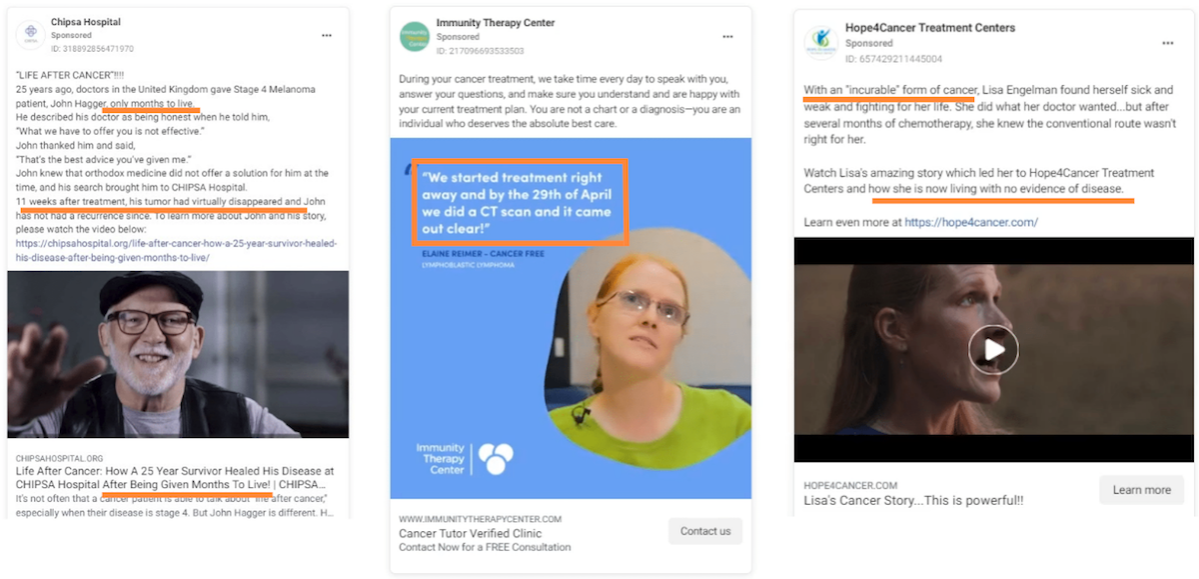
Advertisements featuring testimonials of past clients allegedly cured of cancer.

Across the advertisements, 54.2% (n=168) featured 1 or more people with cancer who received treatment from a provider and spoke about their experience, either about the impact of alternative treatment on their cancer diagnosis or their experience with the advertiser. Figure 4 displays examples of advertisements depicting supposed clients speaking to services received as improving or curing their cancer. Many contain specific references to being cancer-free after receiving treatment with an alternative provider. An advertisement from Envita Medical Centres includes a statement from a person depicted as a patient stating, “I came in here with stage 4 colorectal cancer, [and] I’m leaving cancer free.” Another advertisement reads, “My oncologist didn’t believe it was possible to cure my cancer. Thanks to Immunity Therapy Center, I proved him wrong!”

Alternative cancer providers marketed holistic approaches to healing in 39% (n=121) of advertisements, including emotional health, addressing trauma, and other factors impacting a person’s ability to treat and fight their cancer. Providers emphasized incorporating psychological wellness into their treatments. For example, a Budwig Medical Centre advertisement states, “It is a treatment for your physical body, but it also a treatment for your soul—it is an emotional and psychological treatment.”

Approximately 26.1% (n=81) of advertisements featured language conveying care about their patients’ well-being, often emphasizing the relationship they want/do build with their patients. For example, an Immunity Therapy Centre advertisement states, “Our knowledgeable and loving team invests time in developing relationships that bless everyone involved.” Other promotions highlight apparent vulnerable, caring moments between staff and patients. A Brio-Medical advertisement reads, “Dr Larry was there [while patient crying], and he hugged me, and I knew after that it was going to be great.” Last, advertisements emphasize treating clients not just as another case. A Budwig Centre ad states: “You are not a chart or diagnosis—you are an individual who deserves the absolute best care.”

**Figure 5:**
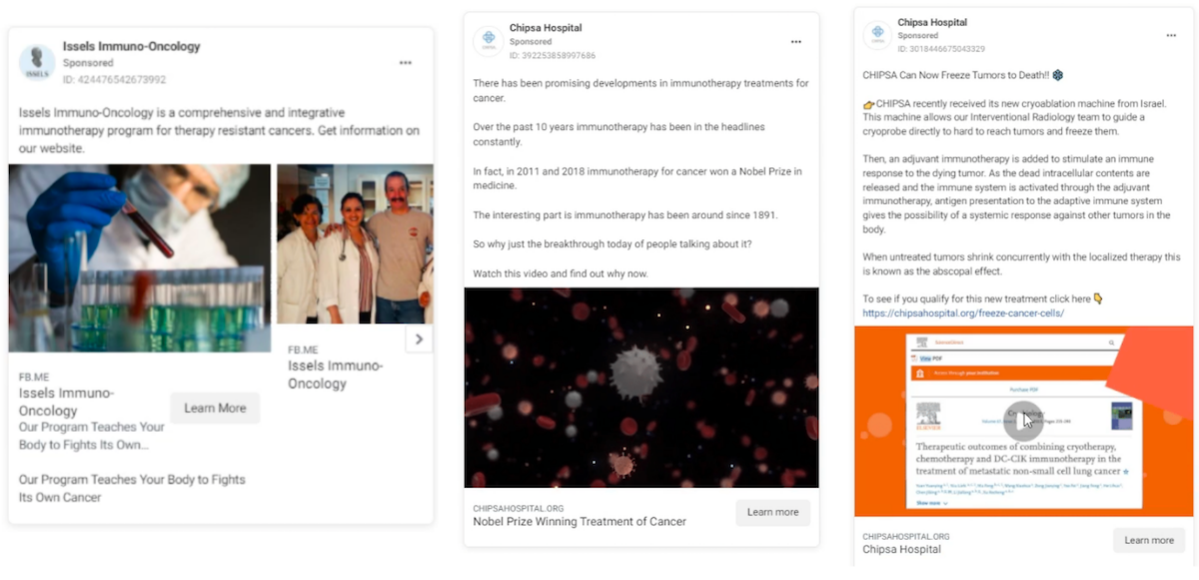
Advertisements referencing provider treatments rooted in science and research.

Providers sought to support the effectiveness or legitimacy of their treatments or approach by referencing rhetoric or imagery related to science, research, evidence, and well-known science-related organizations or institutions in 23.2% (n=72) of advertisements. Cues for coding included the terms “research-based,” “Harvard medical,” “NASA,” “new research,” “Nobel prize,” “proven,” “published,” “scientific evidence,” “researched,” “scientifically proven,” and related terms. Here, providers gave little to no details about the research mentioned and included images of cells or other biological processes with no context (see Figure 5). In many cases, unproven, disproven, or experimental treatments were represented as being supported by research. For example, Brio-Medical states, “Researchers are using vitamin C and oxygen to kill cancer.” Advertisements also included misleading statements about the research quality or implications for specific treatments.

In 20.3% (n=63) of the advertisements, providers represented themselves as keeping up to date and offering the latest technological advances in their cancer treatments and approach. Providers used terminology signaling major innovation, including “groundbreaking,” “breakthrough,” “new,” “paradigm shift,” “state of the art,” and related terms. In some cases, the clinic’s latest advanced technology was used as an appeal. For example, an advertisement from Conners Clinic links to the clinic founder’s “groundbreaking book” on treating cancer. In other cases, advertisements mention the facilities as a “state-of-the-art center.”

Messaging stating that the key to treating cancer is understanding why it developed in the first place was observed in 13.9% (n=43) of advertisements. Here, clinics argue that treating cancer requires identifying and removing the reasons leading to cancer development. For example, an advertisement from Brio-Medical states, “Stop fighting cancer and address the cause by asking why your body is sick.” Most often, clinics recommend making certain lifestyle or diet changes to prevent reoccurrence and promote healing. For example, Conners Clinic recommends a 4-pronged treatment for healing cancer that consists of “cause, nutrition, technology, diet, and detoxification.”

Across the 310 advertisements analyzed, 25.8% (n=80) included 1 or more direct statements signifying the offered cancer treatments at their facility are effective for curative or life-prolonging purposes or that the treatment offered has cured or prolonged life in patients. Example excerpts of these direct statements are included in Textbox 1; note that these are raw text and have not been edited for grammar. We found 78 cancer treatments mentioned in advertisements (Multimedia Appendix 2). The most mentioned treatments or approaches were alternative (n=191, 61.6%) and natural (n=153, 49.4%). Many clinics do not advertise the full range of treatments they offer.

**Example statements signifying that the offered cancer treatment is effective for curative or life-prolonging purposes, or treatment offered has cured or prolonged life in patients.**

**Sample excerpts**

- “From hospice to healed! CHIPSA saves another cancer patient.”
- “It really was just about the 2-week mark where I really had noticeable improvement in how I felt, and my breast lump started shrinking so that was pretty amazing.”
- “Craig was diagnosed with colorectal cancer and came to the Budwig Center in August 2014 to receive treatment pursue the natural approach. Just a year later, in May 2015, the doctors shared with some good news: his cancer had totally disappeared.”
- “Eight years later: Bailey O’Brien shares how she be terminal melanoma at CHIPSA.”
- “Aaron’s stage IV glioblastoma survivor story.”
- “My oncologist didn’t believe It was possible to cure my cancer, thanks to Immunity Therapy Center I proved him wrong!”
- “11 weeks after treatment, his tumor had virtually disappeared and John has not had a recurrence since.”
- “But nearly two years after her initial diagnosis, and treatment at CHIPSA, Amanda is still alive to share her story, and remarkably, she’s cancer free!”
- “Rebecca’s battle with thyroid cancer led her to seek a more integrative approach. She found Verita Life Thailand. Following treatment at our clinic in Bangkok, today, she is cancer-free.”
- “How Michelle overcame breast cancer with immunotherapy based on dendritic cells: ‘I’ve been getting treatments for about a month and there is no evidence of the tumour whatsoever.’”
- “Envita totally saved my life.”
- “I stayed the full 6 weeks just to get all the good therapies and it took me to a place of being cancer free.”
- I came in here with stage 4 colorectal cancer, I’m leaving cancer free.”
- “Find out like I did yesterday that my tumor is gone.”

## Discussion

### Principal Findings

Our results provide evidence that alternative cancer providers are using Meta products to advertise alternative cancer treatments to social media users. Advertisements regularly referenced “alternative” and “natural” approaches to cancer treatments. Imagery and text content emulated evidence-based medical providers and created the impression the treatments were legitimate medical options for cancer. Similarly, advertisements exploited the hope [57] of patients with terminal cancer and poor prognoses by sharing testimonials of past patients who allegedly were cured, had their lives prolonged, or had their quality of life improved. Providers framed their services as filling a gap once conventional medicine runs out of treatment options and sought to differentiate themselves from evidence-based medical providers who delivered a terminal diagnosis by undermining the efficacy of their administered cancer treatments (eg, radiation, chemotherapy) and their care and compassion for their patients.

Providers appealed to prospective patients with cancer through “scienceploitation” [58], which occurs “when popular scientific ideas…are used to take advantage of the social capital associated with them and induce consumer interest in products or services” and can “create misunderstandings and/or posits false connections” [59]. Providers shared narratives of their clinics offering breakthrough, advanced, and scientifically supported services outside the traditional medical scope. In other cases, providers conveyed information about promising scientific treatments, such as immunotherapy, but did not contextualize the inability of the clinic to properly manufacture, administer, and monitor such advanced treatments or correctly explain its evidence base [60]. We also identified scientific language and imagery used in an effort to legitimize unproven therapies and approaches. References and imagery of research, science, specific studies, or experiments in advertisements may distort the viewer’s assessment of how medically accepted the ideas are to which the advertisements were referring. This, in turn, leads to an unfounded belief in the likelihood of treatment success and unnecessary financial and time expenditure.

Meta advertising tools enable alternative clinics to promote and at some level target their advertisements to people with cancer. Prior studies demonstrate how established platform features and tools (groups, timelines, sharing posts) are employed by users and providers to purposefully or inadvertently spread cancer misinformation [4,11,17,61-63]. In difference from such studies, we demonstrate an active element in social media platforms spreading and profiting from misleading medical information. Meta platforms approve advertisements [64], provide targeting options, and earn direct revenue from advertisements. When unproven cancer advertising is found, Meta publicly frames the advertisements against their policies, removes the advertisements, and details interventions to minimize or prevent health misinformation [46,65-67]. Despite removal, alternative cancer treatments can still create new advertisements with disproven claims and use targeting tools. Our results suggest that the case-by-case ad removal after media or user reporting [68] and overreliance upon artificial intelligence by Meta have not addressed nor will be able to address the problem.

Currently, Meta requires an authorization process, “written permissions,” or application procedures for select advertisements (ie, prescription drug advertising, addiction treatment, cryptocurrency, social issues, elections, politics, online pharmacies, online gambling and gaming, and dating) [38]. Expanding the authorization processes to all medical advertisements could potentially limit the dissemination of misleading or exploitative medical advertising identified in this paper. Approval processes should not rely on artificial intelligence tools [69] but instead, be coordinated by qualified medical professionals. Regular audits of approved medical advertisers would likely be necessary to ensure compliance. Strong disincentives, such as banning and reporting advertisers who violate legal and platform policies, may also help limit this harmful practice. Cross-border advertising tools and the reach of advertisements create difficulties [70-73] for country-specific regulatory adherence and enforcement, positioning Meta as the only party with the competency and capability to efficiently police advertisements.

In providing public health recommendations for Meta, the power dynamics between public health researchers and social media platforms must be made transparent and discussed extensively. While we believe these aforementioned recommendations would be effective, they are framed and scaled to what national public health systems have the authority to intervene upon and what is likely to be accepted by Meta [74]. Although a growing body of literature provides recommendations for Meta and other social media platforms to improve public health, we argue that it is important to acknowledge that these proposals likely will not be pursued if they adversely impact social media platform interests or business models [75,76]. With the little power public health researchers and practitioners are availed to change social media policies and processes, recommendations to social media businesses such as Meta are created to appeal to the good nature of platforms or make a case that our suggestions are beneficial to their interests. In most other contexts, appealing to or working with a for-profit industry to improve health in ways against their financial interests is not effective [77] and can hurt public health interests [78], even if case-by-case gains are achieved. This context is emblematic of a larger power dynamic in how social media businesses reinforce their political power, acting as both infrastructure and advertiser, thus both judge and interested party [79,80].

Fully acting upon the issue of misleading advertisements requires examining and confronting the conflict of interest between social media business interests and public health [81]. In the case of misleading health advertisements, this is only a single symptom of a larger infrastructure in pursuit of profit [82,83], and it is at odds with public health objectives. Meta, like most social media businesses, relies on advertisements for revenue. Many advertisements hosted by Meta are harmful to public health or cause direct harm, including those promoting health-harming products [84], dis/misinformation [85], hateful speech [86,87], and other content types. Advertising tools allow invasive targeting [88] for products or messages using data that many users may not know are collected [89] or sold. However, the public health response [90], and indeed Meta’s response, is to accept this system as a status quo and seek ways to improve it incrementally while not recognizing or acknowledging that the business model itself is harmful [91]. It is important to understand the shared responsibility between advertisers and social media platforms, both of whom benefit greatly from deceptive advertising being relayed to the public. This calls for political courage and the use of effective means to avoid such harmful practices.

### Limitations

This study has several limitations. First, the advertisements collected are only a brief snapshot of the advertising of unproven cancer treatments across Meta platforms. The search strategy attempted to identify the most well-known clinics administering unproven medicine; therefore, our results likely undercount the true scale of unproven cancer treatment advertising. The advertisements and clinics identified are also geared toward English-speaking audiences located in North America. Next, we cannot objectively state the testimonial content seen in this study is untrue or that specific cases of cancer were not cured or improved. However, the marketing of curative and life-prolonging testimonials for scientifically unsupported treatment is still dangerous because it provides false hope to patients with advanced or terminal cancer. This study employed a single-coder approach, which may have subjected the data set to the interpretative bias of the coder. However, we took several steps to mitigate this, including cocreating a defined coding frame, test coding, team discussion, and auditing categories with perceived subjectiveness, such as advertising claims of being cured or having life prolonged. Finally, the Meta Ad Library does not provide advertisement viewership data (reach, demographics), advertisement targeting details, conversions, or financial spending information. Thus, we cannot speculate on the viewership impact of the specific advertisements in our sample.

### Conclusion

In this study, we found alternative health providers advertise scientifically unsupported cancer treatments and approaches through paid advertising products on Meta platforms. Advertisements contained 8 distinct strategies to appeal to viewers: advertiser representation as a legitimate medical provider, appealing to persons with limited treatments options, client testimonials, promoting holistic approaches, rhetoric related to science and research, rhetoric pertaining to the latest technology, and focusing treatments on cancer origins and cause. Among the advertisements, 25.8% (n=80) included a direct statement claiming that their treatment can cure or prolong life. The dissemination of advertising poses a serious concern to public health, which may spread misinformation, distrust in evidence-based health care, exploitation of vulnerable groups, unnecessary financial expenditure on unproven treatments, and disengagement from evidence-based cancer treatments. This study also illustrates how Meta advertising tools promote unproven medical therapies and the inadequacy of existing deterrents to prevent misleading medical advertisements. We recommend that Meta introduce a mandatory, human-led authorization process for medically related advertisers before receiving advertising permissions. As social media platforms have historically failed to fully act on such recommendations, we also suggest public health policies be enacted to compel social media companies to better monitor and remove problematic advertisements and ban advertising from companies and individuals with a history of spreading misinformation. Further research should consider an enhanced focus on the conflict of interest between social media platforms advertising products and public health and better characterize the nature and scale of the harm caused by such targeted advertisements.

## Appendix

Multimedia Appendix 1 Clinic profile overview of alternative cancer treatment providers.

Multimedia Appendix 2 Frequency of specific treatments mentioned or displayed in advertisements.

## Abbreviations

CHIPSA: Centro Hospitalario Internacional del Pacifico, SA
FDA: Food and Drug Administration

## Data Availability

The coded data and media files are available upon reasonable request to the corresponding author.
