# Supplementary material for: Advertising Alternative Cancer Treatments and Approaches on Meta Social Media Platforms: Content Analysis
Source: JMIR Infodemiology. 2023 May 31;3:e43548. doi: 10.2196/43548 (PMC10267786; doi:10.2196/43548)
Supplement: Multimedia Appendix 1 [file infodemiology_v3i1e43548_app1.docx]

**Supplementary File 1. Clinic Profile Overview of Alternative Cancer Treatment Providers**

| **Clinic Name** | **Total # of Ads** | **Location (Website)** | **Treatments Offered (Website)** | **Provider Degree Qualifications (Website)** |
| --- | --- | --- | --- | --- |
| **Brio-Medical** | 146 | Scottsdale, Arizona | - Acupuncture - Alpha Lipoic Acid IV - Artemisinin IV - Colon hydrotherapy & enemas - Curcumin IV - Detoxification IV Therapy (Chelation) - DMSO IV - Glutathione IV - High Dose Vitamin C IV - Hydration IV - Hydrogen Peroxide Therapy IV - Hyperbaric Oxygen Chamber Therapy - Hyperthermia - Level 1 Nutrient IV (Customized by the doctor) - Level 2 Nutrient IV (Customized by the doctor) - Level 3 Nutrient IV (Customized by the doctor) - Level 4 Nutrient IV (Customized by the doctor) - Localized hyperthermia (Mag-Ray) - Low-Level Light (Prism) - Magnesium IV - Malnutrition IV - Melatonin IV - Mind-Body Emotional Wellness Counselling - Mistletoe IV - Multi Limen (Laser Therapy) - Natural Immunotherapy - Nutrition - Ozone (rectal and bladder insufflation) - Ozone IV - Ozone sauna - Pain IV - PEMF- High Intensity (Pulse) - PEMF- Low Intensity (Bemer) - Phosphatidylcholine IV - Photo Biomodulation Therapy (Halo) - Poly MVA IV - Polyphenol IV - Quercetin IV - Red Light Therapy - Salicinium IV - Sodium Bicarbonate IV - Ultraviolet Blood Irradiation (UVB Therapy Ozonated Blood IV) - Vibrational Therapy | - NMD - MD - RN |
| **Conners Clinic** | 44 | Lake Elmo, Minnesota | - True rife - Ion pro wave foot bath cleanse - Hammer bulb - PEMF - Hyperthermia - IV Vitamin C - Light beam generator - Hyperbaric oxygen therapy | - DPSc - ND - DC |
| **Chipsa Hospital** | 34 | Tijuana, Mexico | - Coley’s toxins - Immunotherapy - Insulin potentiation therapy - Gerson therapy - CK3 - IV Vitamin C - Vitamin K3 - Apatone (Vitamin C & K3) | - MD - ND |
| **Verita Life** | 23 | Bangkok, Thailand | - Anti-angiogenic therapy - Biological treatment - Herbal cancer therapy - IPT - Local body hyperthermia - Metabolic treatment - Metronomic therapy - Dendritic cell therapy - Natural killer cell therapy - Peptide therapy - Whole body hyperthermia - Anti-cancer diet - Chelation therapy - Colonic therapy - Lymphatic therapy - Ozone therapy | - MD - Nurses (unspecified) - Nutritional experts |
| **Budwig Center** | 14 | Málaga, Spain | - Footbath (lymph and kidney detox) - Full body hyperthermia - Matrix med - Heat pack application on kidney/liver and bladder - Life bio-zapper device - Cell sonic - Reset chair - Four in one - Bio catalytic respiration - Health pulses - Biomagnetic pair therapy - Hyperbaric oxygen therapy - Phytotherapy - Diet - Naturopathy | - PhD - Naturopath (unspecified) - Anthroposophic physician (unspecified) - Expert in bioenergetic acupuncture and moxibustion - Vega and Galvanic skin response therapist |
| **Immucura** | 14 | Málaga, Spain | - Dendritic cell therapy - Immunotherapy - Macrophage activation therapy - Natural killer cell therapy - Nanothermia - Targeted supplementation | Not listed |
| **Hope4Cancer Treatment Centers** | 12 | Tijuana, Mexico | - AARSOTA Bioimmunotherapy - AIC Calcium Therapy - Behavioral, Emotional, & Spiritual Therapy - Biological Dentistry - Coffee Enema - Customized Nutritional Supplementation - Enzyme Therapy - Full Body Hyperthermia - Full Spectrum Nutrition Therapy - Helixor - Herbs (Personalized) - Hyperbaric Oxygen Therapy - Immune Power Plus - Immunoimagery - INDIBA Local Hyperthermia - Juicing - Microbial Herbs & Medications - Near Infrared Lamp Therapy - Near Infrared Sauna Therapy - Nutritional IVs - Ozone Therapy - Photo Dynamic Therapy Plus - PolyMVA Supplementation - Pulsed Electro-Magnetic Field (PEMF) Therapy - Salgena Immunotherapy - Salvus Immunotherapy - Sono-Photo Dynamic Therapy - Sunivera Immunotherapy - Ultraviolet Blood Irradiation - Vibrational Therapy - Vitamin B-17 IV Therapy - Vitamin C IV Therapy | - MD - ND - Nutritionist(unspecified) |
| **Immunity Therapy Center** | 12 | Tijuana, Mexico | - Cartilage (Bovine and Shark) IV - Graviola IV Therapy - Hyperbaric Oxygen Therapy - Intratumoral Immunotherapy - Mononuclear Cell Activation - Psycho-Spiritual Therapy - Virtual Reality Meditation Therapy - Natural Whole Body Hyperthermia Cancer Treatment - Localized Hyperthermia - Halo therapy - Sono dynamic therapy - Laser cancer therapy - Insulin potentiation therapy - DMSO potentiation therapy - Rife machine therapy - Biomagentic cancer therapy - Apatone IV treatment - Immunotherapy - Dendritic cell cancer vaccine - Viral anticancer vaccine treatment - Autologous stem cell therapy - Enzyme therapy - Oxygen therapies - Vitamin and mineral supplementation - IV curcumin therapy - B17 therapy - Regenerative cell cancer therapy - Salinomycin IV - Mag ray - Chelation - Detoxification - Leukocytes activated by interleukin - Specific killer cell vaccine - Intraperitoneal perfusion hyperthermia - Cryoablation therapy - Radiofrequency ablation | - MD - Nurse (unspecified) - Nutritionist |
| **Envita Medical Centers** | 6 | Scottsdale, Arizona | - Genetically Targeted Fractionated Chemotherapy (GTFC) - Chemo Immuno Precision Injections (CIPI™) | - MD - ND - DO - DO(h) - MD (h) - Pharmacists (unspecified) - Nurse (unspecified) |
| **Dayspring Cancer Clinic** | 4 | Scottsdale, Arizona | - Colon hydrotherapy - IV Vitamin C - 3 Bromopyruvate - Evox therapy - Blue scorpion venom - Colloidal silver - Life vessel therapy - Oxygen therapy - Chelation therapy - Ketogenic diet - Budwig protocol - Myers cocktail – Vitamin C - Cellsonic therapy - Cannabis - Cannabidiol - Prayer - Neo7 peptide therapy | - NMD - MD - RDN - LNA - LNC - Phlebotomist |
| **Issels Immuno-Oncology** | 1 | Tijuana, Mexico | - Immunotherapy - Dendritic cell therapy - Natural killer cell therapy and T-cells - Autologous dendritic cell cancer vaccine - Prostate cancer vaccine - Lymphokine-Activated Killer cells - Activated natural killer cells - Systematic hyperthermia - Immunobiologic core treatment program | - ND - Unspecified doctor training |

***Information retrieved from provider websites on Feb 8^th^ and 9^th^ 2022**
