# Supplementary material for: Advertising Alternative Cancer Treatments and Approaches on Meta Social Media Platforms: Content Analysis
Source: JMIR Infodemiology. 2023 May 31;3:e43548. doi: 10.2196/43548 (PMC10267786; doi:10.2196/43548)
Supplement: Multimedia Appendix 2 [file infodemiology_v3i1e43548_app2.docx]

**Supplementary File 2. Frequency of Specific Treatments Mentioned or Displayed in Ads**

| **Treatment** | **Code Frequency** | **%** |
| --- | --- | --- |
| Alternative treatment | 191 | 61.61 |
| Natural treatment | 153 | 49.35 |
| Immunotherapy | 30 | 9.68 |
| Nutraceuticals | 18 | 5.81 |
| Coley's therapy | 12 | 3.87 |
| Vitamin C (IV or not) | 11 | 3.55 |
| Budwig protocol | 7 | 2.26 |
| Hyperthermia | 6 | 1.94 |
| Adjuvant | 5 | 1.61 |
| Curcumin | 5 | 1.61 |
| Low dose chemotherapy | 5 | 1.61 |
| Cryoablation | 5 | 1.61 |
| Apatone (vitamin C and K3) | 4 | 1.29 |
| IV vitamin K3 | 4 | 1.29 |
| Homeopathic | 4 | 1.29 |
| Hydration | 4 | 1.29 |
| Par biomagnetic sessions | 3 | 0.97 |
| GSR scan | 3 | 0.97 |
| Vega test | 3 | 0.97 |
| IPT | 3 | 0.97 |
| Detoxification | 2 | 0.65 |
| Chemo agents | 2 | 0.65 |
| Oxygen | 2 | 0.65 |
| Metronomic therapy | 2 | 0.65 |
| Quercetin | 2 | 0.65 |
| Herbal therapy | 2 | 0.65 |
| High dose vitamin C | 2 | 0.65 |
| Arpwave therapy | 1 | 0.32 |
| Halo biophotonic therapy | 1 | 0.32 |
| Pulse electromagnetic fields | 1 | 0.32 |
| Vitamins (unspecified) | 1 | 0.32 |
| UV blood irradiation | 1 | 0.32 |
| Cold laser therapy | 1 | 0.32 |
| Nutrition | 1 | 0.32 |
| Rife | 1 | 0.32 |
| Cancer vaccine - cryotherapy + immunotherapy | 1 | 0.32 |
| Ozone | 1 | 0.32 |
| Hercules multi-laser therapy | 1 | 0.32 |
| Bemer | 1 | 0.32 |
| IV nutrient therapy | 1 | 0.32 |
| Gerson therapy | 1 | 0.32 |
